# Indents

**Published:** 1911-04-15

**Authors:** 


### INDENTS.

**☞ Brief Articles of Technical or Scientific Value will receive notice and comment. Contributions invited.**

*Mouth Wash, Astringent.*

| | |
|---|---|
| Tincture of Myrrh | 1 dr. |
| Oil of Wintergreen | 1¼ dr. |
| Alcohol | 2½ oz. |
| Water | 1½ oz. |
| Zinc Chlorid | 8 gr. |

This may be used full strength in prophylaxis treatment; when prescribed for patients' use, direct a few drops in a little water, sufficient to make the water a little cloudy. Being an alcoholic solution it will precipitate a little in water. It should never be used strong enough to irritate the tongue when taken into the mouth as a mouth wash.—Dr. Grace Rogers, *Dominion Journal.*

*Sulphurous Acid in Dentistry.* Sulphurous acid has been suggested as a useful application in pyorrhea, painted over the teeth after the mechanical removal of deposits; and as a mouth wash, 10 to 20 drops of 60 per cent. acid to be diluted in a small glass of water. Thus diluted it is said to have no bad effects on the teeth, and to have a beneficial effect upon the soft tissues. A prosthetic piece left over night in enough water to cover it, to which have been added 20 to 30 drops of the acid, and carefully cleaned with water in the morning, will be quite free from odor. It brightens the color of vulcanite, while doing it no injury.—J. K. Pedley, *Dental Cosmos.*

*The Contact Point.* No matter how well a filling may be made, how perfect may the cavity preparation, how well the filling may be condensed, if the contour of the tooth is not properly reproduced, the inter-proximal space preserved, or reproduced if it has been lost, the filling is not a success and will not give the patient the comfort that he has a right to expect and demand. I have very frequently removed fillings for no other reason than that the contact point had been so poorly made, the filling so poorly contoured that the patient was in constant misery every time he ate fibrous food. In order to obtain the proper contour there must be a separation in order to restore the portion of the tooth that has been lost by the process of decay, this space may be obtained in any way that suits the operator, either immediate with the separator, or previous separation by any means that he may prefer. In cases of very great loss of the mesio-distal diameter it will take considerable time to restore the necessary space, but it can be done if the operator perseveres. It may be necessary for the patient to wear a gutta-percha plug for months before it is accomplished, but the result will more than compensate the trouble. The fiiling should be finished as perfectly as possibly with a contact point and not a contact plane. All lines should slope to the contact point and so leave the embrasurers out of the area of danger. If this is properly done there will be a good interproximal space.—Rr. Conzetta, *in Review*.

*Pulp and root Treatment.* The method of rational treatment of pulps and roots as indicated by Fischer may be summed up as follows: Every pulp and root treatment necessitates an exact diagnosis, which is made by means of personal anamnesis together with objective symptoms as gained by the application of the induction current or of skiagraphy. Healthy persons with resistant tissue offer a favorable prognosis for the preservation of accidentally exposed or slightly irritated pulps, the cavity to be disinfected with a mixture of chloform and carbolic acid, the pulp capped with Fletcher's cement, and the tooth filled. In feeble and sickly persons the pulp should be devitalized, even if only slightly irritated, with a paste of the following formula: Arsenous oxid and novocain, 4.0 of each; iodoform and thymol, 0.5 of each; crystallized carbolic acid and glycerin, enough to make a paste.

After thoroughly mixing this paste, enough raw silk threads are added, and stirred until the paste has been completely taken up. This prevents a settling of the heavy arsenous oxid, securing uniformity of the medicinal effect. The pulps of all incisors, upper and lower canines, and those in lingual root canal, of upper molars and distal root canals of lower molars should be extirpated, especially in youthful patients. In persons of more advanced age, also in the deciduous set, the pulps in all teeth should be amputated without making an effort at total extirpation. Gangrenous root-canals are dressed with frequently repeated dressings of tricresol and formaline, 2:1, until all phenomena of acute inflammation have disappeared, and are then filled. Amputated pulp horns, as well as empty root-canals, no matter whether pulpitis or ganarene has been present, are covered or filled with a tricresol-formalin paste. For amputated pulps the following paste is also used by the author: Novocain and thymol, thoroughly mixed, 40 per cent. formalin aa 4.0 white vaselin 12.00; this is mixed with zinc oxid into a firm pill, laid into the pulp chamber, and with a pledget of cotton pressed firmly upon the pulp sections. If the pulp horns have been entirely extirpated, this same preparation may be applied for filling the root-canals. The roots thus filled are sealed with annealed asbestos wool and cement. For the final filling amalgam, gold, or porcelain may be used, but not cement. Multirooted teeth with hopelessly gangrenous pulps are often best extracted under local anesthesia induced by the following solution: Novocain 1.0, alypin 1.0, sodium chlorid 0.92, thymol 0.02, aqua destillata 100.0 from two to three drops of a 1 : 1000 solution of synthetic suprarenin to be added to 2 ccm. of the anesthetic solution. The tooth is washed in warm physiologic salt solution, held with a sterile napkin, opened and disinfected with tincture of iodin, and filled with an iodoform bougie and cement. Granulations around the apical portion of the root are burred away or resected, and after about fifteen minutes' work the tooth is ready to be reimplanted, thus prolonging its life for years.—*Cosmos.*

